# Complete Genome Sequence of Bacillus amyloliquefaciens subsp. *plantarum* CC178, a Phyllosphere Bacterium Antagonistic to Plant Pathogenic Fungi

**DOI:** 10.1128/genomeA.01368-14

**Published:** 2015-01-08

**Authors:** Byung-Yong Kim, Sang-Yeob Lee, Jae-Hyung Ahn, Jaekyeong Song, Wan-Gyu Kim, Hang-Yeon Weon

**Affiliations:** a ChunLab Inc., Seoul National University, Seoul, Republic of Korea; b Agricultural Microbiology Division, National Academy of Agricultural Science, Rural Development Administration, Wanju, Republic of Korea

## Abstract

Bacillus amyloliquefaciens subsp. *plantarum* strain CC178 is a phyllosphere bacterium with antagonistic activity against a wide range of plant fungal pathogens. The genome of strain CC178 is 3,916,828 bp in size and harbors 3,972 genes. Six giant gene clusters are dedicated to the nonribosomal synthesis of antimicrobial polypeptides and polyketides.

## GENOME ANNOUNCEMENT

Bacillus amyloliquefaciens is subdivided into two subspecies—B. amyloliquefaciens subsp. amyloliquefaciens and B. amyloliquefaciens subsp. *plantarum*—based on whole-genome comparisons with polyphasic taxonomic data (1). The strains belonging to B. amyloliquefaciens subsp. *plantarum* are known for promoting plant growth and for their diverse secondary metabolites (2). For these reasons, they are good candidates for use as biofertilizers or biopesticides to improve crop yield and quality.

Strain CC178, which was isolated from the phyllosphere of cucumber to find biocontrol agents that demonstrate antifungal activity, was identified as a B. amyloliquefaciens strain by 16S rRNA gene sequencing and physiological and biochemical analyses. The strain suppresses a broad spectrum of pathogenic fungi, including Fusarium oxysporum, *Phytophthora capsici*, *Rhizoctonia solani*, and Sclerotinia sclerotiorum (unpublished data). Here, we analyzed the genome sequence of the strain to explore the genomic features responsible for its effectiveness as a biocontrol agent.

Whole-genome sequencing of strain CC178 was performed with a combined strategy of Roche/454 and Solexa sequencing. A total of 39,947,992 reads were generated to reach a depth of 1,027-fold coverage with an Illumina Solexa Genome Analyzer IIx. To construct scaffolds, Roche/454 paired-end libraries containing 8-kb inserts were constructed, and 233,396 paired-end reads were generated using the GS FLX system, giving 17.9-fold coverage of the genome. A total of 97.3% of the reads were assembled into 4 large scaffolds, including 117 nonredundant contigs, using Newbler version 2.8 (Roche Applied Science, Penzberg, Germany). Most of the gaps within the scaffolds were filled by PCR primer walking. Gaps between scaffolds were filled by custom primer walking and long-distance PCR amplification followed by DNA sequencing with an ABI 3730xL sequencer (NICEM, Seoul, Republic of Korea). Gene prediction was carried out using Glimmer version 3.02 (3) and the Clusters of Orthologous Groups (COG) and SEED databases (4); rRNA and tRNA genes were identified by utilizing RNAmmer version 1.2 (5) and tRNAscan-SE version 1.23 (6), respectively.

The complete genome sequence of the strain was characterized by a circular chromosome of 3,916,828 bp with a 46.5% G+C content without plasmids. A total of 3,972 coding DNA sequences, 86 tRNA genes, and 9 rRNA operons were predicted. The average nucleotide identity was calculated using JSpecies (7). The genome of strain CC178 was found to be closely related to that of B. amyloliquefaciens subsp. *plantarum* FZB42^T^, with an average nucleotide identity of 99.9%. Thus, it is most likely a strain of B. amyloliquefaciens subsp. *plantarum*.

The strain CC178 genome also contained six giant gene clusters devoted to the synthesis of antimicrobial peptides and polyketides by nonribosomal polypeptide synthetases (NRPS) and polyketide synthases (PKS). The clusters were highly similar to those found in the genome of FZB42^T^ (1). The availability of the complete genome sequence provides insights into the genomic basis of its antifungal mechanism and facilitates the exploration of genomic traits related to plant growth and health.

### Nucleotide sequence accession number.

The complete genome sequence of strain CC178 has been deposited at DDBJ/EMBL/GenBank under the accession number CP006845.
